# MiR‐17‐5p promotes cancer cell proliferation and tumorigenesis in nasopharyngeal carcinoma by targeting p21

**DOI:** 10.1002/cam4.863

**Published:** 2016-10-24

**Authors:** Chun Chen, Zenghong Lu, Jie Yang, Weichao Hao, Yujuan Qin, Huiyan Wang, Congying Xie, Raoying Xie

**Affiliations:** ^1^ Department of OrthopaedicsThe First Affiliated HospitalWenzhou Medical UniversityWenzhou325000China; ^2^ Department of OncologyThe First Affiliated HospitalGannan Medical CollegeGanzhou341000China; ^3^ Cancer Research InstituteSouthern Medical UniversityGuangzhou510515China; ^4^ Department of Radiation and ChemotherapyThe First Affiliated HospitalWenzhou Medical UniversityWenzhou325000China

**Keywords:** Cell proliferation, miR‐17‐5p, nasopharyngeal carcinoma, p21, poor prognosis

## Abstract

MicroRNAs (miRNAs) may act as either tumor suppressors or oncogenes in various types of cancers. Previous studies have indicated that miR‐17‐5p is involved in the initiation and development of human tumors. However, its mechanism and function in nasopharyngeal carcinoma (NPC) remain largely unclear. In this study, we evaluated the expression profiles of miR‐17‐5p and *p21* in NPC cell lines and tissues by quantitative real‐time PCR (qRT‐PCR). For the analysis, we have established a stable overexpression or depletion of miR‐17‐5p NPC cell lines for analyzing the effects of cell proliferation by MTT, colony formation, and cell cycle assay. A nude mice xenograft model was used to verify the tumor growth in vivo. MiR‐17‐5p was overexpressed, whereas the expression of *p21* was downregulated in NPC cell lines and tissues. The miR‐17‐5p expression level was inversely correlated with the *p21* mRNA level in NPC samples. Furthermore, analysis of 2^−ΔΔCt^ value in 81 NPC patients suggested that the elevated expression level of miR‐17‐5p or the downregulated expression level of *p21* was significantly correlated with tumor size (T classification) and tumor stage, and Kaplan–Meier survival analysis revealed a correlation between miR‐17‐5p or *p21* expression level and overall survival times in 81 NPC patients. MiR‐17‐5p promoted cell growth in vivo and in vitro by directly targeting p21. Our results indicate that miR‐17‐5p can promote the occurrence of NPC and it may serve as a potential novel diagnostic maker or therapeutic target for NPC in the future.

## Introduction

Nasopharyngeal carcinoma (NPC) is an Epstein–Barr virus (EBV)‐associated malignant tumor that occurs in nasopharyngeal epithelial tissues. NPC has the highest incidence rate in Southern China and Southeast Asia, represents a significant disease burden, and has an incidence of approximately 2/10,000 per year in endemic areas 1. Genetic susceptibility 2, EBV infection 3, and chemical carcinogens 4 have been implicated in the pathogenesis of NPC. However, its pathogenesis is still not very clear. To improve the cure rate of NPC, we need to further clarify its molecular mechanism.

MicroRNAs (miRNAs) are conserved, 21‐ to 23‐nucleotide long noncoding RNAs; they repress the target gene expression mainly by binding to its 3′ UTR, thus making it silenced or overexpressed 5, 6. Available studies have shown that they are very important class of tumor cellular functions regulators, including proliferation 7, invasion and metastasis 8, differentiation 9, and apoptosis 10, and also participate in the initiation and development of NPC. It has been reported that miR‐17‐5p is involved in different kinds of cancers, including pancreatic 11, gastric 12, bladder 13, hepatocellular 14, breast 15, lung 16, and cervical cancers 17, and plays an important role in the regulation of cell cycle 18. MiRNA microarray data show that the expression levels of miR‐17‐5p were increased in NPC tissues compared with the control group 19, 20; and the expression of miR‐17‐5p was upregulated in the serum of patients with NPC 21. However, the mechanism and function of miR‐17‐5p is not very clear in NPC.

The discovery of *p21* gene plays an important role in cell cycle control and tumor development. It is considered as the tumor suppressor, and inhibits cyclin‐dependent kinases (CDKs) complex activity 22. MiR‐17 has been elucidated to target p21 in other contexts including some cancers. MiR‐17 promotes the developing cortex through suppressing p21 expression for maintaining the neural progenitor pool 23. Overexpression of miR‐17‐5p promotes cell growth in chronic myelogenous leukemia and B‐cell lymphoma cell lines by targeting p21 24. MiR‐17 directly inhibits the p21 expression in myeloid leukemia 25 and promotes the tumor growth by suppressing p21 in synovial sarcomas 26. In addition, some previous studies showed that the deletion of *p21* led to poor survival prognosis in some cancers 27, 28, 29, 30. However, the role of the loss of *p21* has not been conclusively determined in the clinical outcome of NPC.

In our study, the results show that miR‐17‐5p is highly expressed in NPC tissues compared to that of noncancerous controls. MiR‐17‐5p induced cell proliferation and tumorigenesis by targeting p21. It is confirmed to be as a tumor suppressor in NPC. MiR‐17‐5p may be a new therapeutic target for improving the cure rate of NPC.

## Materials and Methods

### Tissue samples

The tissues samples were obtained from patients who did not receive chemoradiotherapy. All participants signed the consent forms before carrying out this research and received the approval from the Human Ethics Committee of Gannan Medical College.

### Cell culture

Normal nasopharyngeal epithelial cells (NP69) and NPC cell lines (6‐10B, 5‐8F, CNE2, CNE1, HNE1, HONE1, and C666‐1) were cultured in RPMI‐1640 medium containing 10% fetal bovine serum (FBS; HyClone), incubated at 37°C, and 5% CO_2_.

### RNA isolation, reverse transcription, and qRT‐PCR

The Trizol reagent, iScript cDNA synthesis kit, and SYBR PrimeScript miRNA RT‐PCR kit were purchased from TaKaRa Bio. The procedures of RNA isolation, reverse transcription, and qRT‐PCR were carried out according to the manufacturer's protocol and our previous publications 31. The primer sequences used for the amplification of the indicated genes in this study are listed in Tables S1 and S2. Fold changes were calculated by 2^−ΔΔCt^ method. U6 and GAPDH were used as endogenous control.

### MTT assay, colony formation assay, and cell cycle analysis

The detection procedures are as described in Lyu et al. 32.

### Luciferase assay

The dual‐luciferase reporter assay system kit was purchased from Promega Corporation (Promega, Madison, WI) and the testing procedure was based on the manufacturer's instructions and as described previously 33.

### Western blot analysis

The protocols for western blot were well described previously 34. The primary antibodies against p21, pRb, Rb, E2F1, CCNE1 and CCND1 (Cell Signaling Technology, Boston), and *β*‐actin (Santa Cruz Biotechnology, Dallas) were used in this study.

### Immunohistochemistry

The staining procedure was performed according to the manufacturer recommendations protocol and the detailed steps were described in Luo et al. 35.

### Animal studies

We purchased the nude mice from Guangdong Experimental Animal Center. All experiments on mice in this study were conducted by the Laboratory Animals Care and Use Committee of Southern Medical University. We made all our efforts to minimize any possible suffering of the animals. As described previously 36, cells (1 × 10^6^) were resuspended with 100 *μ*L Matrigel and transplanted into the flanks of 5‐week‐old nude mice (*n* = 5). The length (L) and width (W) of tumors were measured every 3 days and calculated using the formula: Volume (mm^3^) = L × W^2^ × 0.5. All nude mice were executed after 3 weeks; we carefully collected tumor samples from mice and weighed them.

### Statistical analysis

The SPSS 16.0 software was used for all statistical analyses in this study. Two‐tailed Student's *t* test, chi‐square test, and log‐rank test were used to analyze the data. Data are presented as mean ± SD. *P*s < 0.05 were considered to be statistically significant.

## Results

### MiR‐17‐5p was upregulated in NPC

**Figure 1:**
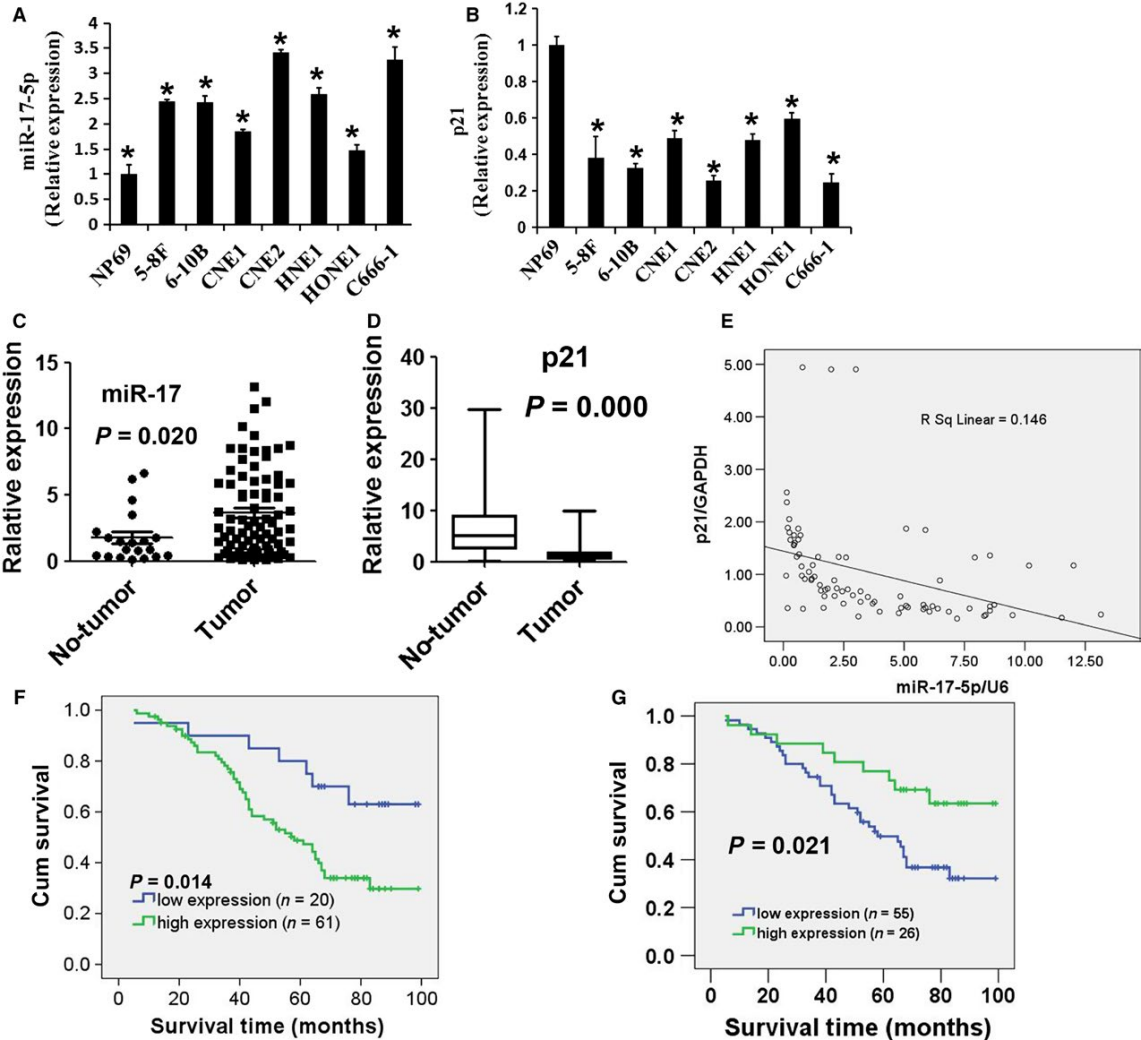
Upregulation of miR‐17‐5p and downregulation of p21 mRNA expression in NPC cell lines and tissues. (A) miR‐17‐5p was overexpressed in seven NPC cell lines in comparison with that of NP69. Values represent mean ± SD, *P < 0.05. (B) p21 mRNA expression was detected by qRT‐PCR in seven NPC cell lines. Values represent mean ± SD, *P < 0.05. (C) The expression of miR‐17‐5p was detected in 81 NPC tissues and 20 nontumor tissues by qRT‐PCR. miR‐17‐5p expression was upregulated in NPC tissues. (D) The expression of p21 was analyzed in 81 NPC tissues and 20 nontumor tissues by qRT‐PCR. (E) Correlation analysis between p21 and miR‐17‐5p in 81 clinical tissue samples. (F, G) Kaplan–Meier survival analysis of overall survival duration in 81 patients according to miR‐17‐5p and p21 gene expression levels. Statistical analysis was conducted using a log‐rank test (*P* < 0.05). NPC, nasopharyngeal carcinoma; mRNA, microRNAs.

The expression level of miR‐17‐5p was examined in 81 NPC samples and 20 no tumor nasopharyngeal epithelial tissues by qRT‐PCR. The test results showed that the expression of miR‐17‐5p was upregulated in NPC compared to normal controls (Fig. 1C). Furthermore, we analyzed the association between clinicopathological factors and miR‐17‐5p or *p21* expression in 81 NPC patients. Although the results showed that a patient's gender, age, lymph node, and distant metastasis had no significant association with miR‐17‐5p and *p21* expression levels, we observed that the elevated expression level of miR‐17‐5p or the downregulated expression level of *p21* was significantly correlated with tumor size (T classification) and tumor stage in NPC patients (Table 1).

**Table 1:** Associations between the clinicopathologic factors and miR‐17‐5p and p21 expression in 81 nasopharyngeal carcinoma patients. (*P*<0.05, the differences were statistically significant)

Variables	*N*	MiR‐17‐5p expression*N* (%)			p21 expression*N* (%)		
		Low	High	*P*	Low	High	*P*
Gender							
Male	58	15 (25.9)	43 (74.1)	0.698	40 (69.0)	18 (31.0)	0.555
Female	23	5 (21.7)	18 (78.3)		15 (61.9)	8 (38.1)	
Age (year)							
>50	43	12 (27.9)	31 (72.1)	0.517	32 (74.4)	11 (25.6)	0.462
<50	38	8 (21.6)	30 (78.4)		23 (60.5)	15 (39.5)	
T classification							
T1–T2	22	13 (59.1)	9 (40.9)	**0.000**	9 (40.9)	13 (59.1)	**0.003**
T3–T4	59	7 (11.7)	52 (88.3)		46 (75.4)	13 (24.6)	
N classification							
N0–N1	35	11 (31.4)	24 (68.6)	0.220	19 (54.3)	16 (45.7)	0.068
N2–N3	46	9 (19.6)	37 (80.4)		36 (73.5)	10 (26.5)	
M classification							
M0	63	12 (19.0)	51 (81.0)	0.867	44 (69.8)	19 (30.2)	0.145
M1	18	8 (17.8)	10 (82.2)		11 (52.4)	7 (47.6)	
Tumor stage							
I–II	60	7 (11.7)	53 (88.3)	**0.000**	46 (80.7)	14 (19.3)	**0.001**
II–IV	21	13 (61.9)	8 (38.1)		9 (56.2)	12 (43.8)	

### The expression of *p21* was negatively correlated with the expression level of miR‐17‐5p

Often, *p21* is misregulated and plays the opposite role in human cancer. It can also act as an oncogene or a tumor suppressor, because its function was depending on cell types and microenvironment 22. The *p21* expression was clearly downregulated in seven NPC cell lines in this study (Fig. 1B), which was confirmed by qRT‐PCR (Fig. 1D). MiR‐17‐5p, belonging to the miR‐17‐92 cluster, was increased in seven NPC cell lines (Fig. 1A) and NPC tissues (Fig. 1C). We also found that *p21* expression was inversely correlated with miR‐17‐5p expression (Fig. 1E), implying that miR‐17‐5p may regulate p21 in NPC. Kaplan–Meier plots were used to evaluate the survival differences according to the miR‐17‐5p and *p21* expression levels. The low expression levels of miR‐17‐5p or high expression levels of *p21* could increase the survival (Fig. 1F; *P* = 0.014) (Fig. 1G; *P* = 0.021). Our results show that the high expression levels of miR‐17‐5p or low expression levels of *p21* are associated with high death rates from NPC patients.

### High expression levels of miR‐17‐5p increased cell proliferation in vitro

**Figure 2:**
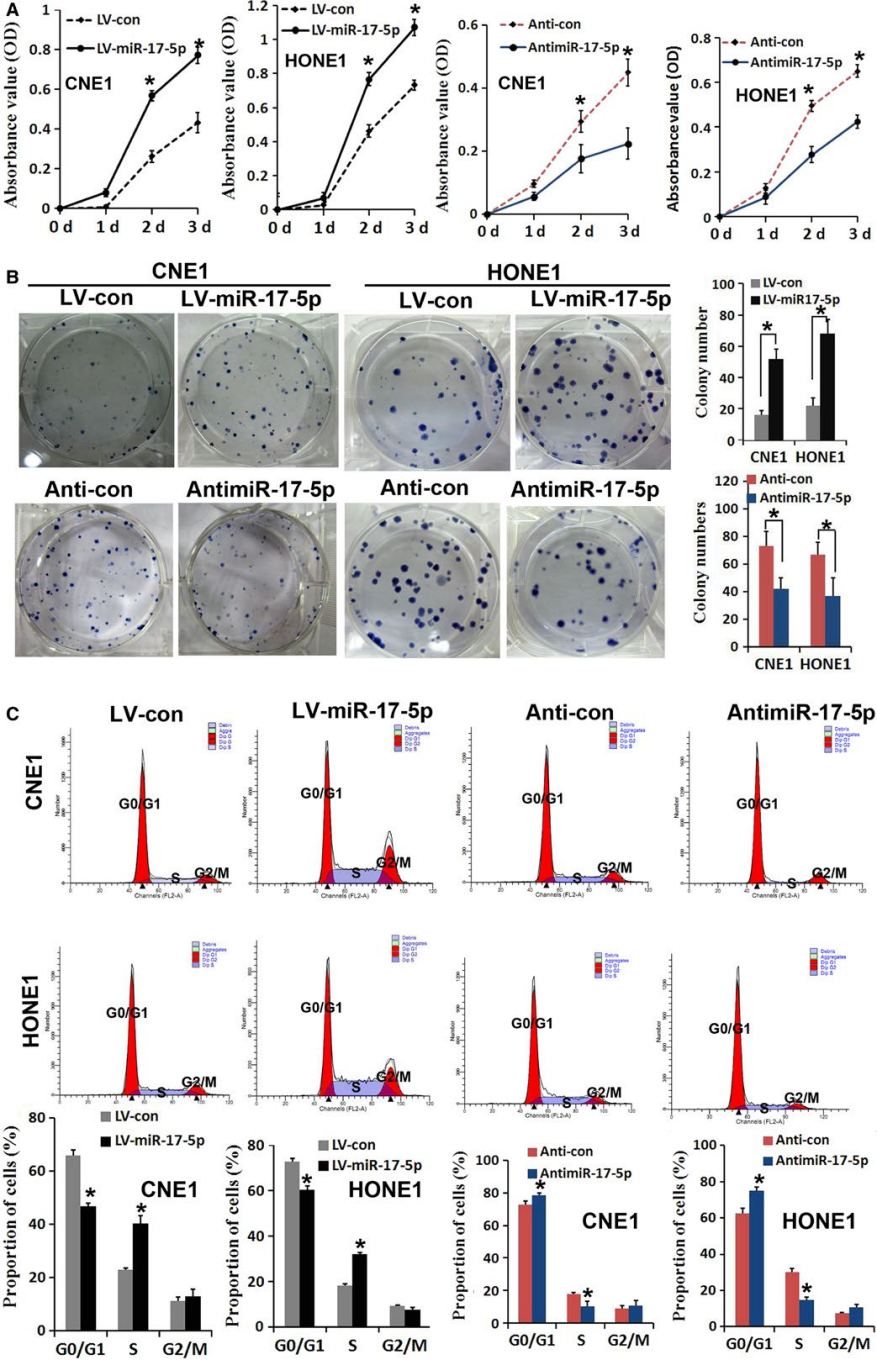
miR‐17‐5p promotes cell proliferation in NPC cell lines. (A) The overexpression of miR‐17‐5p (LV‐miR‐17‐5p) increased the cell viability of CNE1 and HONE1 cells; inhibition expression of miR‐17‐5p (anti‐miR‐17‐5p) decreased the cell viability of CNE1 and HONE1 cells. (B) The colony formation assay was performed in CNE1 and HONE1 cell lines with the overexpression or inhibition expression of miR‐17‐5p. (C) Cell cycle analysis of CNE1 and HONE1 cell lines with stable expression or inhibition of miR‐17‐5p. NPC, nasopharyngeal carcinoma. Values represent mean ± SD, *P < 0.05.

The expression of miR‐17‐5p was examined in seven NPC cell lines (6‐10B, 5‐8F, CNE2, CNE1, HNE1, HONE1, and C666‐1) and normal nasopharyngeal epithelial cells (NP69) by qRT‐PCR. The results indicated that miR‐17‐5p was significantly overexpressed in NPC cell lines compared to that of NP69, and had the lowest expression levels in CNE1 and HONE1 cell lines (Fig. 1A). Therefore, we chose the two cell lines for subsequent experiments. Previous studies indicated that the expression of miR‐17‐5p in NPC was upregulated 20, 21, but the mechanism of miR‐17‐5p has not been elucidated in NPC. Hence, to assess the biological function of miR‐17‐5p, we established both stable upregulation and inhibition expression of miR‐17‐5p in CNE1 and HONE1 cell lines (Fig. S1 and S2). The overexpression of miR‐17‐5p enhanced the proliferative ability of NPC cells, whereas, on the contrary, downregulation of miR‐17‐5p inhibited cell proliferation (Fig. 2A). This viewpoint is further verified by the cloning experiment, the results of which showed that cells with overexpressing miR‐17‐5p formed more colonies than those of the vector control cells, a difference which was statistically significant; the opposite result was obtained with miR‐17‐5p expression inhibition (Fig. 2B). In addition, we examined the DNA profiles of asynchronous populations of cells overexpressing miR‐17‐5p and the vector control NPC cell lines by flow cytometry. A proportion of cells within the S phase in the miR‐17‐5p overexpression NPC cells was higher than the vector control cell lines, and G1/G0 cell ratio decreased in their cell cycle distribution when compared to the vector control cell lines; miR‐17‐5p expression inhibition decreased the proportion of S phase cells (Fig. 2C). The results show that miR‐17‐5p exerts a promoting role on the proliferation of NPC cells.

### miR‐17‐5p directly target p21

**Figure 3:**
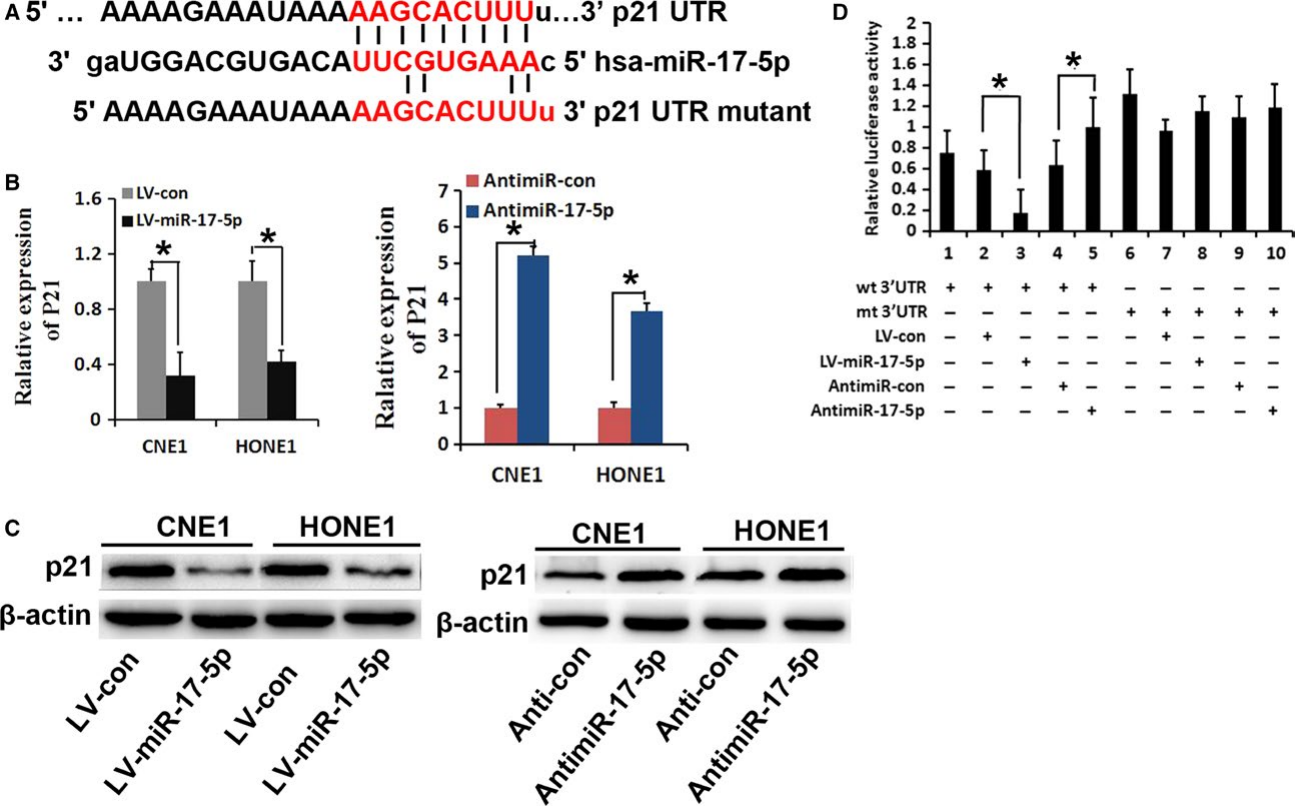
p21 is a target of miR‐17‐5p. (A) A sequence alignment of miR‐17‐5p and the p21 3′ UTR. The seed regions of miR‐17‐5p and the seed‐recognizing sites in the p21 3′ UTR are indicated in red. (B, C) miR‐17‐5p transfection affects p21 expression levels. CNE1 and HONE1 cells were transfected with LV‐miR‐17‐5p, LV‐control, anti‐miR‐17‐5p, or anti‐control. Gene and protein levels were verified by qRT‐PCR and western blot analysis. (D) Luciferase reporter assay of CNE1 cells was performed as described in the Materials and Methods. The bar graph shows the mean ± SD of three independent transfection experiments (*P < 0.05).

To identify that miR‐17‐5p play a role on NPC by targeting p21, we performed a bioinformatic analysis by using microRNA.org and TargetScan Human 7.0. The miR‐17‐5p seed sequence had a complementary match with the 3′ UTR of *p21* (Fig. 3A). As expected, a decrease in p21 mRNA and protein expression was observed after the overexpression of miR‐17‐5p (Fig. 3B and C). In contrast, miR‐17‐5p inhibition expression increased obviously p21 expression level (Fig. 3B and C). In order to further validate that miR‐17‐5p affects the biological behavior of NPC cells by direct targeting p21, the binding sequence of *p21* 3′ UTR (wt 3′ UTR) and the mutant sequence (mt 3′ UTR) were subcloned into a luciferase reporter vector (Fig. 3D). CNE1 cells were then cotransfected with miR‐17‐5p mimic and wt or mt 3′ UTR vector. The results showed luciferase activity significantly decreased when CNE1 was transfected with a miR‐17‐5p mimic compared with the control vector (Fig. 3D, lanes 2 and 3; *P* < 0.01). The activity had no statistical significance by simultaneous transfection with the mt 3′ UTR vector (Fig. 3D, lanes 7 and 8). Moreover, there was an approximately 1.5‐fold increase in luciferase activity when cotransfection with anti‐miR‐17‐5p and wt 3′ UTR vector in CNE1 cells (Fig. 3D, lanes 4 and 5; *P* < 0.01). In summary, the results above strongly suggest that p21 gene is one of the direct targets of miR‐17‐5p in NPC cells.

### MiR‐17‐5p induces cell growth by targeting p21

**Figure 4:**
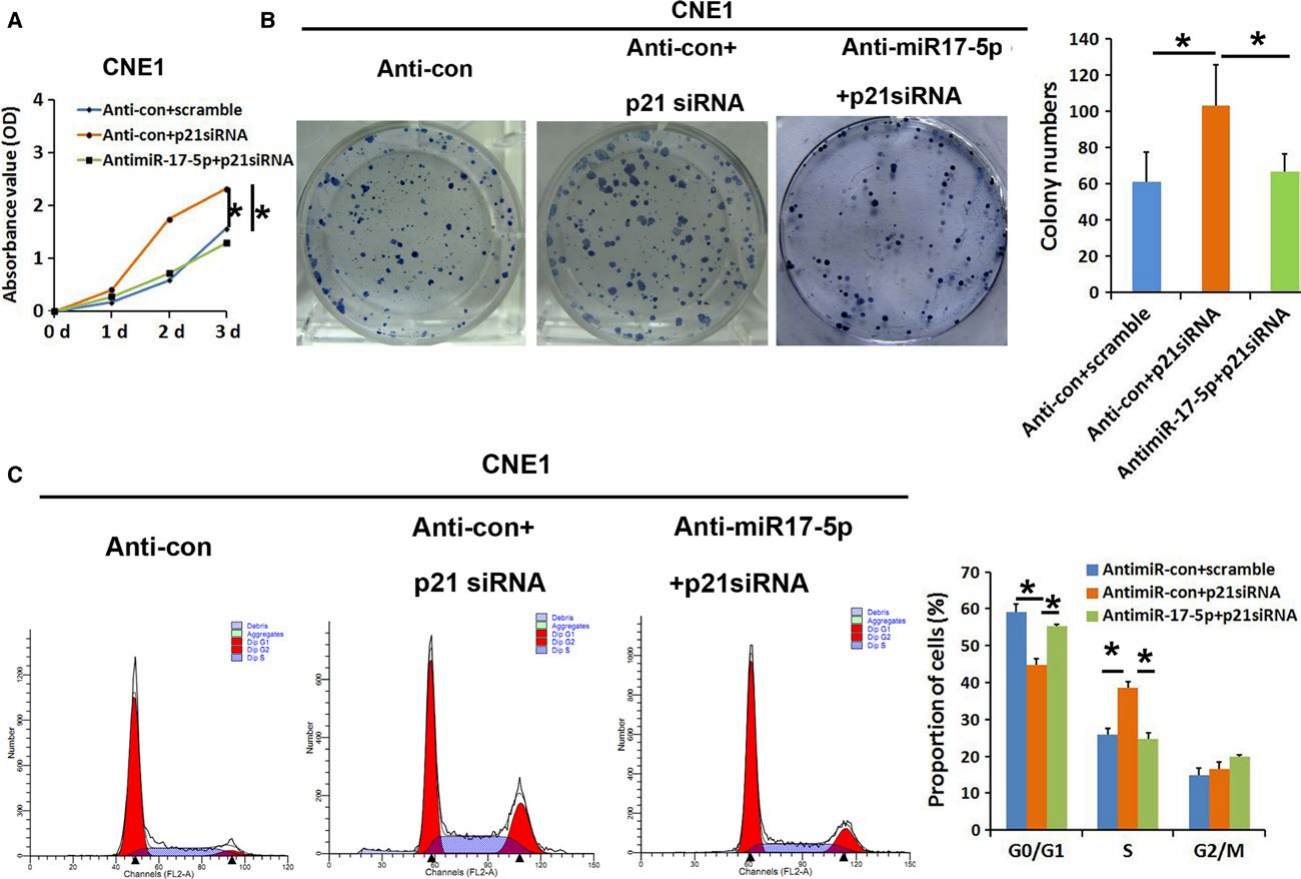
Knockdown of p21 promotes cell growth of NPC cells. (A–C) Effects of p21 siRNA and miR‐17‐5p/p21 siRNA on cell proliferation were evaluated using an MTT assay, the colony formation assay, and cell cycle analysis. The data represent the mean values ± SD from three independent experiments (*P < 0.05). NPC, nasopharyngeal carcinoma.

To elucidate whether the miR‐17‐5p‐accelerated cell proliferation was mediated by the repression of *p21* in NPC cells, we performed loss of function and gain of function studies. The *p21* was silenced in NPC cells. As expected, knockdown of p21 enhanced cell growth in CNE1 cells by using MTT assay, colony formation assay, and cell cycle analysis (Fig. 4A–C).

### MiR‐17‐5p increases cell growth through the p21‐pRb pathway

**Figure 5:**
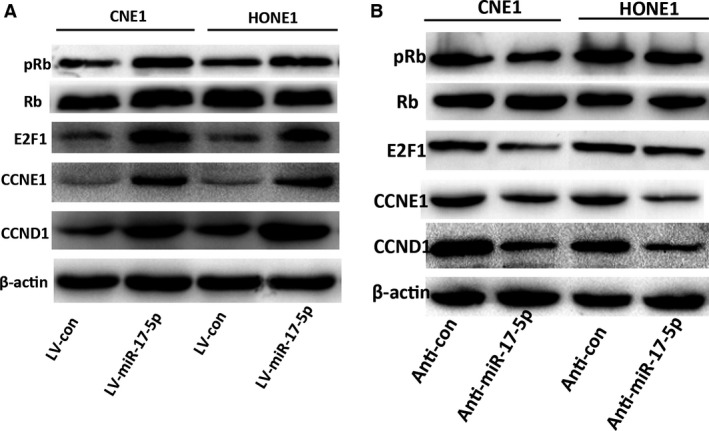
miR‐17‐5p promotes cell proliferation through the p21‐pRb pathway. Western blot analysis of the expression levels of pRb, Rb, E2F1, CCNE1, and CCND1 in CNE1 and HONE1 cells with (A) overexpression of miR‐17‐5p and (B) inhibition expression of miR‐17‐5p.

Subsequently, we checked the expression alterations of some important proteins related to cell cycle and cell proliferation in CNE1 and HONE1 cells with the overexpression or inhibition of miR‐17‐5p by western blotting. Notably, the overexpression of miR‐17‐5p increased the protein expression levels of phosphorylated‐Rb protein (pRb), E2F1, cyclin E (CCNE1), and cyclin D (CCND1) in CNE1 and HONE1 cells (Fig. 5A). Conversely, the inhibition of miR‐17‐5p expression was accompanied by downregulation of pRb, E2F1, CCNE1, and CCND1 in CNE1 and HONE1 cells (Fig. 5B). These data suggest that some cell proliferation and cell cycle gene proteins changed in NPC cells by miR‐17‐5p‐mediated p21 downregulation.

### MiR‐17‐5p promotes tumor growth in vivo

**Figure 6:**
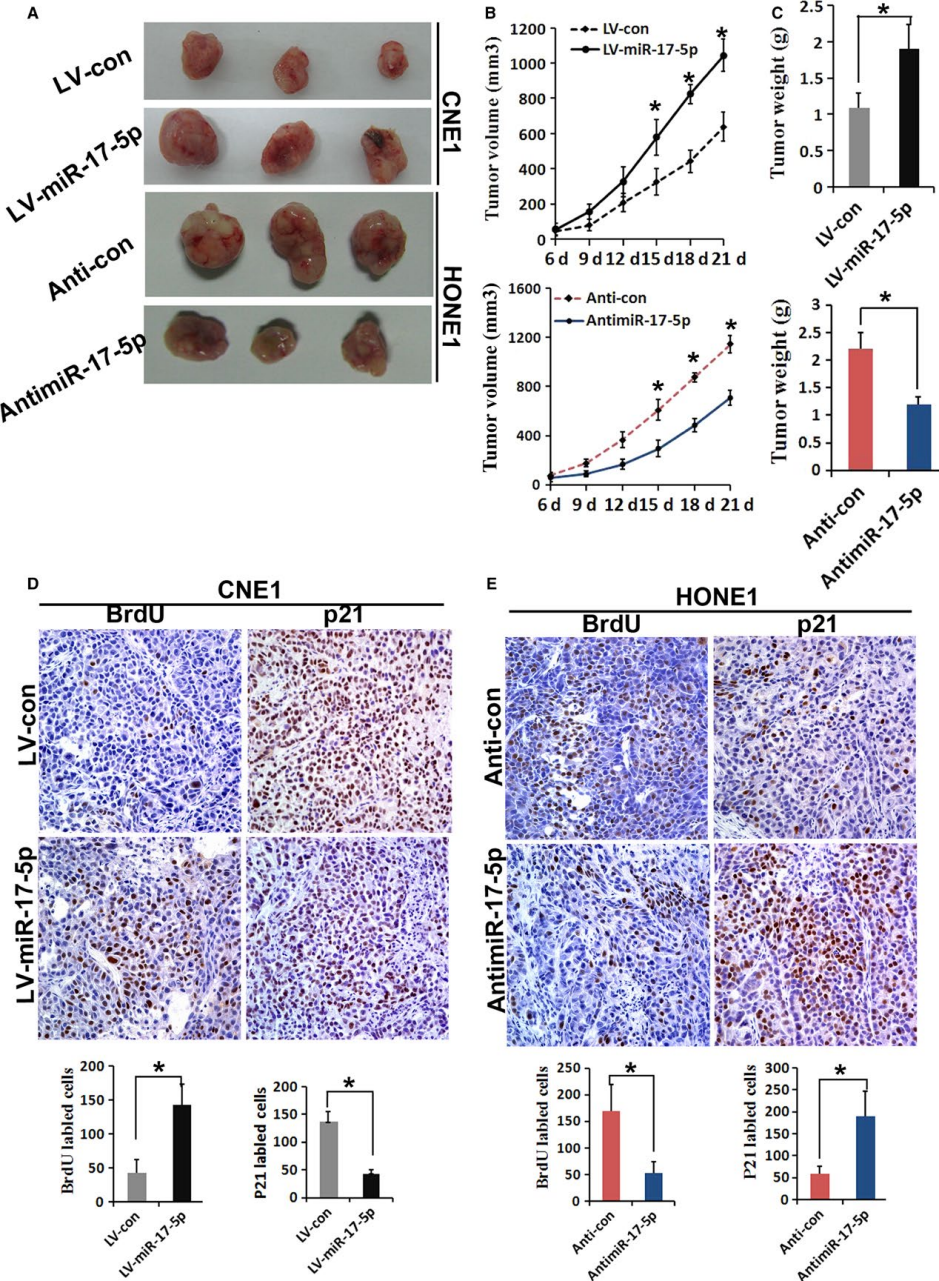
miR‐17‐5p promotes tumor growth in vivo. Tumor formation in nude mice 21 days after injection with CNE1‐LV‐miR‐17‐5p, CNE1‐LV‐con, HONE1‐anti‐miR‐17‐5p, and HONE1‐anti‐miR‐con. (B) Growth curve drawn by measuring tumor volumes at the indicated times. (C) Weight of xenograft tumors. (D) IHC analysis of CNE1‐LV‐miR‐17‐5p‐and CNE1‐LV‐con‐generated tumors 3 weeks after injection. Sections derived from tumors were incubated with anti‐BrdU and anti‐p21 antibody. Representative fields are shown (×400). Of each generated tumor, five fields were randomly selected according to semiquantitative scales. BrdU‐ and p21‐positive cells were counted by three independent experienced pathologists. The bar graph shows average expression levels of BrdU and p21 of CNE1‐LV‐miR‐17‐5p‐ and CNE1‐LV‐con‐generated tumors. (E) IHC analysis of HONE1‐anti‐miR‐17‐5p‐ and HONE1‐anti‐con‐generated tumors 3 weeks after injection. BrdU and p21 were detected using the same method of detecting CNE1 cells tumors. IHC, Immunohistochemistry. Values represent mean ± SD, *P < 0.05.

To further confirm the important role of miR‐17‐5p in NPC, a nude mouse xenograft model was used in this study. The mice were transplanted with CNE1 cells with overexpressing miR‐17‐5p and scramble control or HONE1 cells with inhibited expression of miR‐17‐5p and antiscramble control. After 21 days, tumor sizes from overexpressing miR‐17‐5p cells were significantly larger than those from the scrambled control cells (Fig. 6A). Furthermore, overexpression of miR‐17‐5p significantly increased the volume (Fig. 6B) and weight of xenograft tumor (Fig. 6C). Immunohistochemistry (IHC) analysis results showed that p21 protein expression was significantly reduced in the tumor samples induced by CNE1‐miR‐17‐5p cells (Fig. 6D). Consistently, the upregulation of BrdU was observed in miR‐17‐5p overexpressing tumors (Fig. 6D).

Conversely, tumors with downregulation of miR‐17‐5p were smaller than those of mice transfected with the scrambled control cells (Fig. 6A), and the xenograft tumor volume and weight decreased (Fig. 6B and C). IHC revealed enhanced p21 expression in tumors in which miR‐17‐5p was inhibited (Fig. 6E). Consistently, BrdU downregulation was observed in tumors formed from miR‐17‐5p‐inhibited HONE1 cells (Fig. 6E). These results indicate that miR‐17‐5p can promote tumor growth by targeting p21 in vivo.

## Discussion

The miR‐17‐92 gene cluster and its paralogs have an established oncogenic function 37, and each component of the cluster is also reported to be overexpressed in a variety of tumors, both hematopoietic and solid tumors including breast, lung, pancreas, colon, prostate, and stomach cancer 38. Campisi indicated that this cluster played a very important role in regulating cell cycle network and tumor occurrence 39. The previous studies implied that the miR‐17‐92 gene cluster and its paralogs were as important regulatory factors on NPC, and acted as an oncogene in NPC. MiR‐17‐5p, belonging to the miR‐17‐92 cluster, participates in a variety of cancers. It is well known that the functions of miRNAs in the initiation and development of cancer are very complicated, they can be as both a tumor suppressor gene and an oncogene. For example, miR‐17‐5p is upregulated and an oncogene in pancreatic cancer 11, but it could inhibit cell growth and promote cell apoptosis in cervical cancer 17. Previous studies suggest that miR‐17‐5p is an important regulator of cell cycle 13, 18. Although some studies indicate that the expression level of miR‐17‐5p is upregulated in NPC 20, 21, the mechanism of regulating tumor progression in NPC remains unclear. In this study, miR‐17‐5p obviously promoted NPC cells proliferation, but had no effect on invasion and metastasis (Fig. S3). The *p21* gene, a negative regulator of the cell cycle, was detected and downregulated in cell lines by qRT‐PCR in our pilot experiment. The previous studies showed that miR‐17‐5p could target the p21 in some cancers 24, 25, 26, such as myeloid leukemia and synovial sarcoma. Therefore, we focused on the p21 gene and speculated that miR‐17‐5p may play an important role through targeting p21 in NPC. In addition, cell apoptosis was not obviously observed in our pilot experiment, although previous reports also suggested that p53, a very important tumor suppressor gene, is a target gene of miR‐17‐5p 40 and represses miR‐17‐92 gene cluster expression 41; we did not verify it in our manuscript, but we will further do more validation in the future. Finally, our results demonstrated that miR‐17‐5p acted as an oncogene in NPC via directly targeting p21.

The p21 protein has been defined as a cell cycle inhibitor. However, the clinical significance in NPC is not clear. The high expression level of *p21* in leukemic stem cells can be as a protective factor for DNA damage and promotes cell proliferation by cell cycle inhibition 42. In addition, experiments on colorectal cancer cell lines have suggested that p21 gene induced resistance to chemotherapy 43. The loss of p21 was associated with poor survival prognosis in rectal cancer 29. If we increase p21 expression, the cancers have a better disease‐free survival by increasing tumor cell cycle inhibition. Our results show that miR‐17‐5p directly downregulates p21 expression by binding to its 3′ UTR gene in human NPC cells and tissues. We conclude that miR‐17‐5p promoted NPC cells growth by targeting p21. Therefore, downregulation of miR‐17‐5p might contribute to prolonging the NPC patient's life by maintaining a high level of p21.

In conclusion, the upregulated expression of miR‐17‐5p was observed in NPC tissue samples. We described in detail the function of miR‐17‐5p in NPC. Moreover, we showed that miR‐17‐5p increased the proliferation rate of NPC cells by targeting p21 mRNA. High expression of miR‐17‐5p or low expression level of *p21* was associated with poor prognosis of NPC patients. We believe that miR‐17‐5p may serve as a biomarker in NPC and provide new possibility for drugs development and targeted therapy of NPC in the future.

## Conflict of Interest

None declared.

## Supporting information


**Figure S1.** MiR‐17‐5p was stably overexpressed in CNE1 and HONE1 cells.

**Figure. S2.** MiR‐17‐5p was stably inhibited in CNE1 and HONE1 cells.

**Figure S3.** Cell migration and invasion have no change in miR‐17‐5p‐expressing CNE2 cells and inhibition of miR‐17‐5p‐expressing HONE1 cells.

**Table S1.** Primers for qRT‐PCR analysis of miR‐17‐5p.

**Table S2.** Primers for qRT‐PCR analysis.
