# Supplementary material for: MiR‐17‐5p promotes cancer cell proliferation and tumorigenesis in nasopharyngeal carcinoma by targeting p21
Source: Cancer Med. 2016 Oct 24;5(12):3489–99. doi: 10.1002/cam4.863 (PMC5224848; doi:10.1002/cam4.863)
Supplement: Supplementary file 2 — Figure. S2. MiR‐17‐5p was stably inhibited in CNE1 and HONE1 cells. [file CAM4-5-3489-s002.docx]

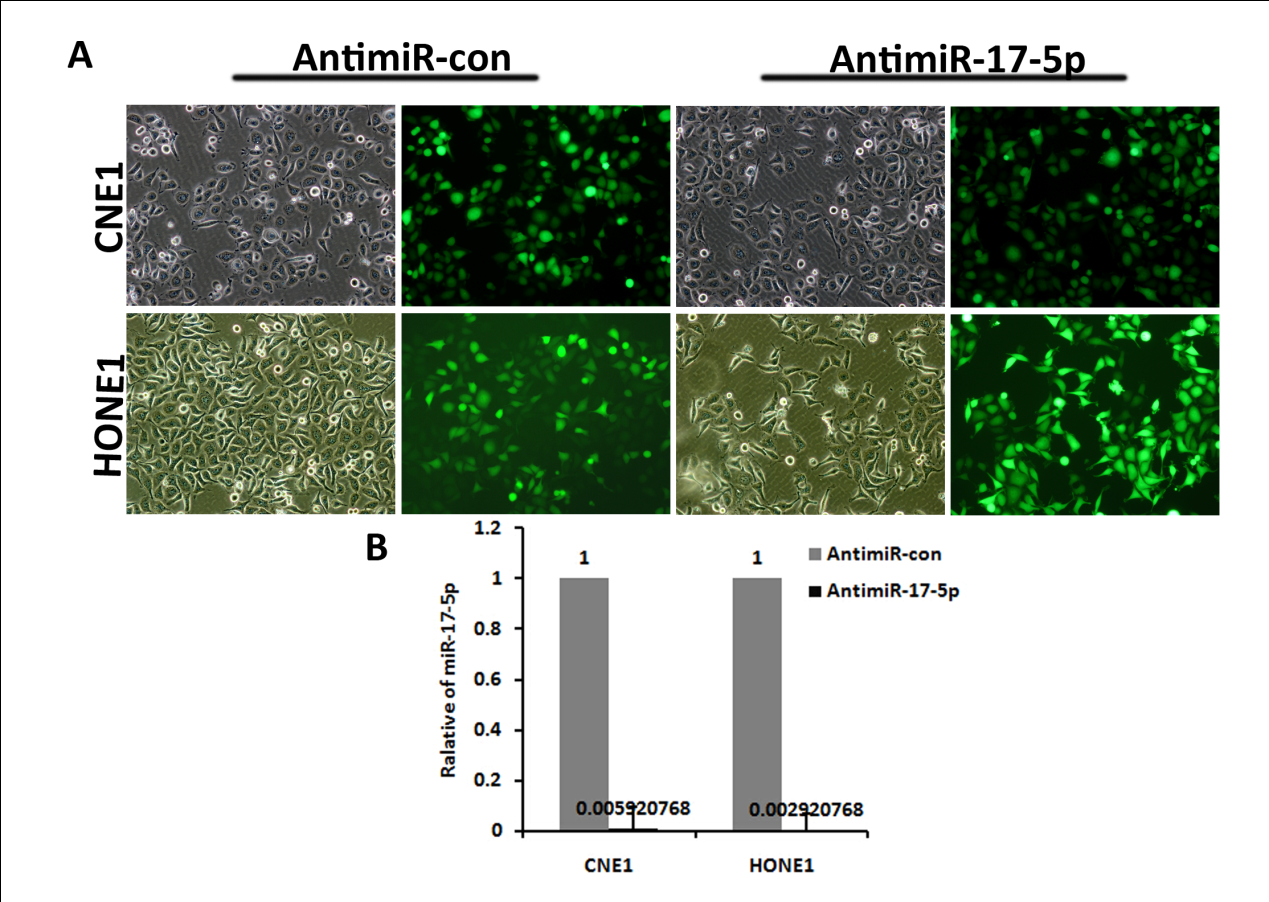


**Fig. S2 MiR-17-5p was stably inhibited in CNE1 and HONE1 cells.**

(A) EGFP assay under inverted fluorescence microscopy for EGFP expression in CNE1 and HONE1 cells harboring EGFP gene. (B) The detection of miR-17-5p expression in CNE1 and HONE1 cells by qRT-PCR.
