# Supplementary material for: MiR‐17‐5p promotes cancer cell proliferation and tumorigenesis in nasopharyngeal carcinoma by targeting p21
Source: Cancer Med. 2016 Oct 24;5(12):3489–99. doi: 10.1002/cam4.863 (PMC5224848; doi:10.1002/cam4.863)
Supplement: Supplementary file 3 — Figure S3. Cell migration and invasion have no change in miR‐17‐5p‐expressing CNE2 cells and inhibition of miR‐17‐5p‐expressing HONE1 cells. [file CAM4-5-3489-s003.docx]

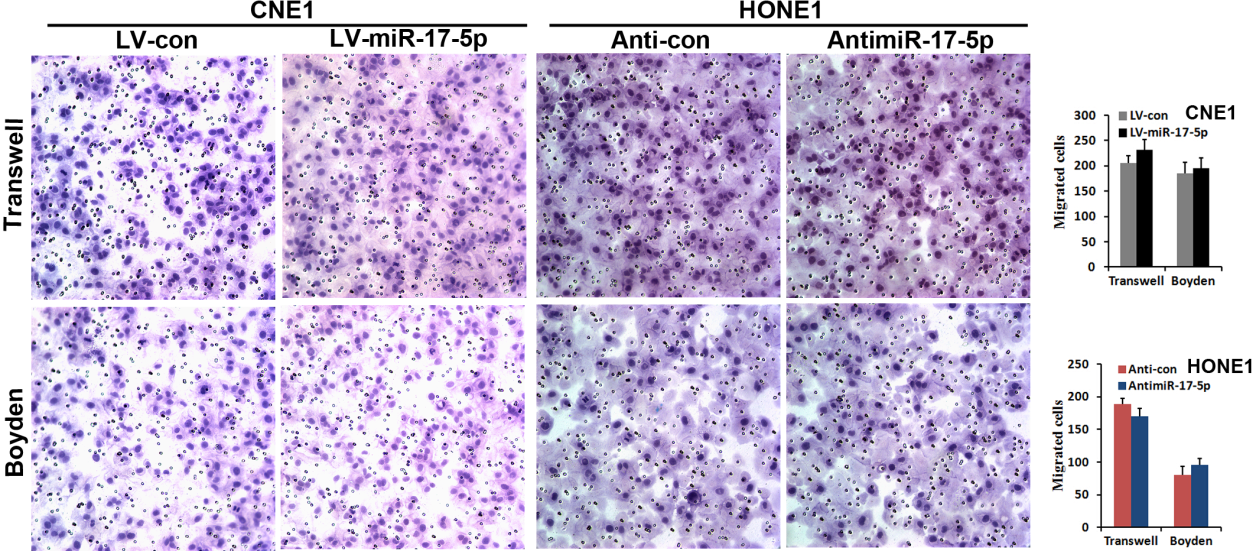


**Fig. S3 Cell migration & invasion have no change in miR-17-5p-expressing CNE2 cells and inhibition of miR-17-5p expression HONE1 cells.**

The motile and invasive properties of miR-17-5p-expressing CNE2 cells and inhibition of miR-17-5p expression HONE1 cells were analyzed by in vitro migration assay using transwell chamber and by in vitro invasion assay using a Matrigel-coated Boyden chamber, respectively. Migrated cells were plotted as the average number of cells per field of view from 3 different experiments, as described in Methods.
