# Supplementary material for: MiR‐17‐5p promotes cancer cell proliferation and tumorigenesis in nasopharyngeal carcinoma by targeting p21
Source: Cancer Med. 2016 Oct 24;5(12):3489–99. doi: 10.1002/cam4.863 (PMC5224848; doi:10.1002/cam4.863)
Supplement: Supplementary file 4 — Table S1. Primers for qRT‐PCR analysis of miR‐17‐5p. [file CAM4-5-3489-s004.docx]

**Table S1 Primers for qRT-PCR analysis of miR-17-5p**

| **Primer name** | **Primer sequence** |
| --- | --- |
| hmiR-17-5p RT primer | GTCGTATCCAGTGCAGGGTCCGAGGTATTCGCACTGGATACGACCTACC |
| hmiR-17-5p forward primer | TGCAAAGTGCTTACAGTGCAG |
| hmiR-17-5p reverse primer | GTGCAGGGTCCGAGGTATTC |
| RNU6-1 RT primer | AACGCTTCACGAATTTGCGT |
| RNU6-1 forward primer | CTCGCTTCGGCAGCACA |
| RNU6-1 reverse primer | AACGCTTCACGAATTTGCGT |
