# Supplementary material for: MiR‐17‐5p promotes cancer cell proliferation and tumorigenesis in nasopharyngeal carcinoma by targeting p21
Source: Cancer Med. 2016 Oct 24;5(12):3489–99. doi: 10.1002/cam4.863 (PMC5224848; doi:10.1002/cam4.863)
Supplement: Supplementary file 5 — Table S2. Primers for qRT‐PCR analysis. [file CAM4-5-3489-s005.docx]

**Table S2 Primers for qRT-PCR analysis**

| **Gene** | **Forward primer (5’-3’)** | **Reverse primer (5’-3’)** |
| --- | --- | --- |
| P21 | GGGATGAGTTGGGAGGAGG | CGGCGTTTGGAGTGGTAG |
| GAPDH | ACCCAGAAGACTGTGGATGG | TCTAGACGGCAGGTCAGGTC |
